# Breaking bad: necroptosis in the pathogenesis of gastrointestinal diseases

**DOI:** 10.3389/fimmu.2023.1203903

**Published:** 2023-06-20

**Authors:** Jay V. Patankar, Marvin Bubeck, Miguel Gonzalez Acera, Christoph Becker

**Affiliations:** ^1^ Department of Medicine 1, University of Erlangen-Nuremberg, Erlangen, Germany; ^2^ Deutsches Zentrum Immuntherapie (DZI), Erlangen, Germany

**Keywords:** necroptosis, inflammatory bowel disease, MLKL, caspase 8, RIPK3, therapeutic

## Abstract

A delicate balance between programmed cell death and proliferation of intestinal epithelial cells (IEC) exists in the gut to maintain homeostasis. Homeostatic cell death programs such as anoikis and apoptosis ensure the replacement of dead epithelia without overt immune activation. In infectious and chronic inflammatory diseases of the gut, this balance is invariably disturbed by increased levels of pathologic cell death. Pathological forms of cell death such as necroptosis trigger immune activation barrier dysfunction, and perpetuation of inflammation. A leaky and inflamed gut can thus become a cause of persistent low-grade inflammation and cell death in other organs of the gastrointestinal (GI) tract, such as the liver and the pancreas. In this review, we focus on the advances in the molecular and cellular understanding of programmed necrosis (necroptosis) in tissues of the GI tract. In this review, we will first introduce the reader to the basic molecular aspects of the necroptosis machinery and discuss the pathways leading to necroptosis in the GI system. We then highlight the clinical significance of the preclinical findings and finally evaluate the different therapeutic approaches that attempt to target necroptosis against various GI diseases. Finally, we review the recent advances in understanding the biological functions of the molecules involved in necroptosis and the potential side effects that may occur due to their systemic inhibition. This review is intended to introduce the reader to the core concepts of pathological necroptotic cell death, the signaling pathways involved, its immuno-pathological implications, and its relevance to GI diseases. Further advances in our ability to control the extent of pathological necroptosis will provide better therapeutic opportunities against currently intractable GI and other diseases.

## 1 Introduction

The GI tract is lined by a single layer of columnar epithelial cells, the enterocytes, which protect the host by forming a tight barrier to the external environment. The intestinal epithelial cells (IEC) form one of the largest barrier surfaces with the absorptive surface of the adult human small intestine alone having an estimated surface area of 250m^2^, which is in constant contact with a myriad of environmental and microbial components (1, 2). This barrier is crucial in mediating a healthy host-commensal symbiosis and therefore an uncontrolled breach of this barrier can quickly become a cause of microbial dysbiosis (1, 2). Dysbiosis can allow opportunistic pathogens access to the portal circulation increasing the likelihood of further inflammation in organs such as the liver and the pancreas. It has long been speculated that a defective gut barrier in humans may be a precursor to liver diseases such as steatohepatitis and cholangitis. The hypothesis that a leaky gut may be a harbinger of long-term inflammatory changes in distant tissues has been demonstrated using preclinical models and has shown positive association in patients (3–7).

Like other contact surfaces of the body such as the skin, the IECs are constantly shedding and sloughing into the intestinal lumen, a process regulated by physiological cell death programs such as apoptosis and anoikis. These coordinated cell death programs ensure that the tight junctions of neighboring non-dying cells seal the gap as the dying IEC exits the epithelial lining. To counteract this tight epithelial barrier, opportunistic pathogens have evolved mechanisms to induce cell death of IEC in order to breach the barrier to gain systemic access. For example, effector proteins such as *Cif* released by the enteropathogenic *E. coli* induce IEC apoptosis and the enterotoxin released by *C. perfringens* binds to the tight junction protein claudin-4, loosening the barrier and eventually resulting in IEC death (8, 9). The damaged IECs must be replaced to restore homeostasis, and one of the protective host mechanisms that accomplishes this is the secretion of TNF by macrophages and other myeloid cells that are activated by invading bacteria. However, TNF is a double-edged sword, inducing cell shedding and apoptosis of compromised IECs, but at sufficient concentrations can also trigger cell death of neighboring, healthy IECs. In addition to these effector mechanisms used by the pathogenic bacteria, viruses also employ strategies that inhibit and exploit various components of the programmed cell death machinery. For example, the virulence factor *vIRA* of mouse cytomegalovirus can target the RHIM domain-mediated interactions between key programmed cell death components RIP1, RIP3, and TRIF, whereas the *K13* inhibitor produced by the Kaposi’s sarcoma-associated herpesvirus prevents the activation of caspase-8 (10). Such strategies employed by pathogens induce host proinflammatory responses that eliminate infected cells via natural killer and cytotoxic lymphocytes. However, immune over-activation or failure of immune suppression after pathogen clearance can cause disproportionate cytokine production, wreaking havoc by inducing pathological levels of IEC death, barrier disruption and immune activation (11). Once pathogens gain systemic access, the next target organ via the portal circulation is the liver, which like the gut is a tolerogenic, but an immune privileged organ (12). Pathogen access is mitigated mainly via the liver resident macrophages namely the Kupffer cells, although cholangiocytes remain susceptible to recurrent cytokine insults leading to pathological cell death (13–15). This establishes a vicious cycle of inflammation - cell death - barrier damage - further inflammation, and once such a self-perpetuating vicious cycle is established, it leads to the chronicity of intestinal and hepatic inflammation such as that observed in patients with inflammatory bowel disease (IBD) and primary sclerosing cholangitis (PSC).

Therefore, several therapeutic strategies have been explored that attempt to block the onset of necrosis in the gut. The desired outcome of a successful therapy is to achieve a long-term restoration of the IEC barrier (2). An example of a treatment that targets IEC death pathways and restores the barrier is that of the TNF inhibitors such as infliximab, adalimumab, and etanercept, however not all patients respond to the treatment and some side effects have also been reported (16–19). Therefore, efforts to understand the molecular and cellular complexity of pathologic cell death in the intestine have been a focus of research. Research has been spearheaded by efforts to draw a detailed picture of the intracellular signaling pathways and molecular nodes that regulate different forms of cell death, followed by research in the design and testing of pharmacological agents that effectively inhibit specific components of the programmed cell death machinery. In the following sections, we will discuss the details of the intracellular machinery involved in programmed necrosis, highlight the findings from the preclinical studies, and review the results of clinical trials and the associated challenges in developing interventions that target necroptosis.

## 2 Molecular machinery of necroptosis

### 2.1 Brief background on necroptosis in the GI tract

Since this review focuses on necroptosis, we refer the reader to other excellent reviews detailing the molecular machinery involved in other programmed death modes in cells of the GI tract (20–24). Necroptosis or programmed necrosis is an alternative form of coordinated cell suicide that culminates in lytic necrosis of the cell. The formal adoption of the term necroptosis is attributable to Degterev et al. in their 2005 report on this non-apoptotic form of cell death (25). Under circumstances where the normal execution of apoptosis cannot occur, necroptosis is triggered and, by extension, is the default mode of caspase-independent cell death that occurs in several cell types. **Table 1** summarizes the evidence for necroptosis susceptibility in various tissue parenchymal cells of the GI tract. It is evident that several resident cell types that make up the majority of GI tissues are susceptible to necroptosis with the exception of hepatocytes, which is still under debated (26–35). Interestingly, there are no reports to date demonstrating whether other resident GI cell types including intestinal fibroblasts, smooth muscle cells, and enteric neurons and glia are susceptible to necroptosis. It is important to note that in the context of GI inflammatory diseases such as IBD, the repertoire of some of these cellular subsets is increased, and in some cases may end up completely replacing the parenchymal cells (36, 37). This is exemplified in intestinal fibrosis and during the inflammation-induced thickening of the intestinal muscle wall, hinting at a potential resistance of these cells to undergo necroptosis in a context-dependent manner that has not yet been formally tested. These cell type-specific differences in necroptosis susceptibility emerge due to the unique expression patterns of specific molecular components involved in the necroptosis pathway. In a later part of this section, we will revisit some of the recent findings on the relative expression abundances of the key components of necroptosis in gut IEC and liver hepatocytes cells and discuss how some GI cells are spared from necroptosis even when the necessary conditions are present.

**Table 1:** Various GI cell types in which necroptosis has been investigated.

Cell type	Pathway/Induced by	Detection method		Citation
		Marker/treatment/rescue	Status	
Enterocytes (m)	TNF- CYLD-RIPK1-RIPK3/enterocyte FADD deficiency	RIPK3 *Ripk3 -/-* cleaved caspase 3	elevated protected absent	(26)
Paneth cells (m)	TNF- RIPK1-RIPK3,/enterocyte Caspase 8 deficiency	TUNEL cleaved caspase 3 RIPK3, necrostatin-1	positive absent elevated blocked cell death	(27)
Hepatocytes (m, h)	Epigenetic silencing of Ripk3 protects from necroptosis	pRIPK1 pRIPK3 pMLKL	absent absent absent	(28, 29)
Cholangiocytes (m)	TNF-TAK1-RIPK1-RIPK3	T+S+z Nec1s	Elevated blocked	(30)
Kupffer cells (m)	listeriolysin O - type 1 interferon – IRF3 induced and RIPK1 dependent, but RIPK3 independent.	cleaved Caspase 1 necrostatin-1 necrostatin-1s *Rip3 -/-*	absent blocked cell death blocked cell death no blockade of cell death	(31)
Endothelial cells (lymphatic and capillary) (m)	Inducible endothelial cell Caspase 8 deficiency, enhanced further by TNF	TUNEL+, CD31+ MLKL knockout	elevated rescues the phenotype	(32)
Pancreatic beta cells (m)	TNF- RIPK1-RIPK3	T+S+z GSK872	elevated blocked	(33)
Pancreatic acinar cells (m)	Caerulein or taurolithocholic acid-3-sulfate	PI pMLKL necrostatin-1 *Rip3 -/-*	elevated elevated blocked blocked	(34)

m, mouse; h, human; p, phosphorylated; oligo, oligomeric; Cl, cleaved; EthD, ethidium homodimer; TUNEL, terminal dUTP nick end labeling; T+S+z, recombinant TNF+ Smac mimetic + zVAD-fmk; Nec1, Necrostatin 1; Nec1s, 7-Cl-O-Nec-1 a derivative of Nec1; PI, propidium iodide.

### 2.2 Initiation of the molecular cascades leading to necroptosis

Analogous to extrinsic and intrinsic apoptosis, necroptosis has both extracellular and intracellular activators. Before delving into the details, the readers is referred to **Box 1** for a brief overview of the most important molecular players in necroptosis. Several signals are known to induce necroptosis in susceptible cells including extracellular TNF, TRAIL, FASL, LPS, dsRNA, and intracellular dsDNA, which can be recognized by cells expressing the specific receptors, namely TNFR1, DR4/DR5, FAS, various TLRs, and ZBP1 respectively. Upon ligand binding, the cytosolic domains of the TNFR, FAS, and DR4/DR5, and TLR receptors assemble into unique signalosome complexes. Under conditions of cellular stress, and when the magnitude of the trigger is sufficient to suppress the downstream NF-κB mediated survival queues, CASPASE-8 inhibits RIPK1 and CYLD and activates the effector caspases CASPASE 3/7, leading to apoptotic death. Recent studies have also linked environmental stresses such as heat shock and osmotic shock as inducers of necroptosis (38). In particular, heat shock protein 90, which is activated in hyperthermic conditions, has been implicated in a non-canonical activation of RIPK3 and MLKL during necroptosis in the colorectal cancer cell line HT-29 (39, 40). Furthermore, hyperosmotic stress triggers necroptosis by increasing intracellular pH (41). It is important to note that in all of these triggering conditions, a contextual prerequisite for necroptosis to occur is a defective initiation of apoptotic cascades. This may occur when initiator caspases such as CASPASE-8 are blocked biologically (inherited mutations, virally encoded inhibitors, host factors c-FLIP, or reduced expression) or pharmacologically (10, 42, 43). Furthermore, biological CASPASE-8 blockade was recently shown to occur via phosphorylation of pro-Caspase 8 at Thr265 by p90 ribosomal S6 kinase downstream of the TNFR-PDK1 (44). In such cellular contexts, the formation of a multi-protein signaling scaffold called the “necrosome” occurs, leading to necroptosis. The necrosome is essentially a multi-protein signaling complex containing activated RIPK1, RIPK3, and MLKL, with activated RIPK1recruiting RIPK3 via the homotypic interaction motif (RHIM domain) (45). The importance of the RHIM domain in mediating the interaction between RIPK1 and RIPK3 is underscored by the finding that mutation of the RHIM domain of RIPK3 attenuates necroptosis (46). Lack of CASPASE-8 activity and deubiquitination of RIPK1 by CYLD are also critical for the stability of the complex (47, 48). Another important requirement for the formation of the necrosome is the inhibition of the A20 deubiquitinase, which blocks the progression of necroptosis by modifying the ubiquitination on RIPK3 (48, 49).

**Box 1: Critical nodes regulating necroptosis:**

  ◼ *FADD*: Fas-associated protein with death domain

◼ *CYLD*: Deubiquitinase responsible for deubiquitinating RIPK1 and TRAF2 promoting necroptosis via stabilizing RIPK1 and RIPK3 interaction and blocking TRAF2 mediated inhibition of MLKL

◼ *A20*: Deubiquitinase and ubiquitin ligase responsible for blocking RIPK3 – RIPK1 interaction, as ubiquitinated RIPK3 is required for necroptosis progression

◼ *TAK1*: Transforming growth factor-β (TGF-β)-activated kinase 1 is the MAP kinase kinase kinase family member 7 a crucial signaling kinase which dictates mode of programed cell death downstream of TLR and TNFR.

◼ *CASPASE 8*: Cysteine-aspartate protease (CASPASE) is an initiator caspase downstream of TNF family receptors, death receptors, and FAS receptor. Initiates signaling via FADD and RIPK1 that culminates on the activation of effector caspases such as Caspase 3 and 7.

◼ *CASPASE 10*: An initiator caspase that once activated, cleaves and activates effector caspases. Pro-caspase 10 is activated in a FADD-dependent manner at the TRAIL and CD95 death-inducing signaling complexes, but cannot functionally replace Caspase 8.

◼ *RIPK1*: Receptor-interacting serine/threonine-protein kinase 1 acts downstream of TNF family receptors and death receptors and regulates phosphorylation and formation of signaling scaffolds that control apoptosis and necroptosis. More recently, RIPK1 was shown to also regulate inflammasome function.

◼ *RIPK3*: Receptor-interacting serine/threonine-protein kinase 1 is activated via phosphorylation by RIPK1 and in the absence of CASPASE 8 activity and/or activation of ZBP1, forms the signaling scaffold ‘necrosome’ that phosphorylates and activates MLKL.

◼ *ZBP1*: Z-DNA binding protein 1 is an interferon induced sensor of intracellular DNA and triggers the recruitment and activation of RIPK3 upon binding DNA of intracellular pathogens.

◼ *MLKL*: Mixed lineage kinase domain-like protein, is phosphorylated and activated by RIPK3. Some reports have indicated that further MLKL activation may require actions of other kinases including TAM and CAMK2. Upon full activation, MLKL undergoes oligomerization and inserts into the plasma membrane via its N-terminal helix bundle that binds to phosphatidyl inositol phosphates.

### 2.3 Formation of the core necrosome

Phosphorylation of RIPK1 at multiple sites in its intermediate domain is one of the most upstream events in necrosome formation and is mediated by the kinase activity of TAK1, NIK, and the autophosphorylation of mouse and human RIPK1 at Ser166 (50, 51). On the other hand, several kinases including MK2, IKKα/β, TBK1/IKKϵ, phosphorylate and inhibit mouse and human RIPK1 activity via the addition of inhibitory phosphates at Ser320, Ser321, Ser336, and Ser25 (52–56). Interestingly, another study suggested that TAK1-dependent phosphorylation of RIPK1 at Ser321 is essential for its association with FADD and the execution of RIPK1-dependent apoptosis (57). The authors used genetic and pharmacological ablation of TAK1 to demonstrate its critical involvement in phosphorylation of Ser321, challenging the inhibitory role of Ser321 on RIPK1 activity (57). However, a recent report revealed that the non-receptor tyrosine kinases JAK1 and SRC phosphorylate RIPK1 at Tyr384 (human) and Tyr383 (mouse), which are inhibitory phosphates critical in suppressing RIPK1 activation (58). The authors further showed that mice carrying mutations of these Tyr residues that prevent their phosphorylation develop spontaneous systemic inflammation, reinforcing the critical role of the complex post-translational modifications of RIPK1 that regulate this key component of the cell death machinery (58). Recently, we have shown that the activation of the EP4 receptor via endogenous or pharmacological agonists leads to a suppression of RIPK1 phosphorylation at Ser166. We have also implicated TAK1 downstream of the EP4 receptor in this suppressed activation and blocking of necrosome formation under conditions of CASPASE 8 inhibition in IECs (59). These studies highlight the critical role of the complex post-translational modifications of RIPK1 that regulate this key component of the cell death machinery.

Following RIPK3 recruitment, activated RIPK1 in the necrosome phosphorylates and activates RIPK3 at Ser227 (human) and Ser232/Thr231 (mouse) (60, 61). Once activated, RIPK3 undergoes autophosphorylation at Tyr224/Ser227 (human), Tyr231/Ser232 (mouse), and promotes the assembly of heterodimeric oligomers with RIPK1 and with itself via the RHIM domain, forming filamentous fibril-like structures (46, 62). These fibrils can bind thioflavin T and Congo red, and display circular dichroism, and bear structural features of β-amyloids (62). Structurally, activated RIPK1 and RIPK3 form heterotypic alternating stacks that form β-sheets, two of which bind together to form the polymeric fibrils that form the core of the necrosome (63). In addition, the reader is encouraged to note the complexity of the post-translational regulation of RIPK3 as well as that for RIPK1. For example, in certain contexts, RIPK1 is also able to negatively regulate RIPK3 oligomerization and necroptosis, as cells lacking RIPK1 undergo spontaneous RIPK3-mediated death, whereas cells expressing a catalytically inactive form of RIPK1 are protected from cell death (64). Interestingly, RIPK3 activity can be restricted by ubiquitination of the beyond-the-RHIM (BTR) domain, and its TSC1/mTOR - TRIM11-mediated ubiquitination and degradation in IECs alleviates necroptosis and hence colitis (65, 66).

**Box 2: Molecular identification of necroptotic cells: (See limitations in text section 2.4 below)**

Immunohistochemically:

◼ *Co-immunolabelling* – Cleaved Caspase 3/7, terminal deoxyuridine nick end labelling (TUNEL), and pMLKL. Here cells that are TUNEL(+), pMLKL(+), and Cleaved Caspase 3/7 (-) are considered necroptotic.

Molecular Biological:

◼ *Western blotting* – pRIPK3 (+), pMLKL (+), Cleaved Caspase 3/7 (-)

◼ *Immuno co-precipitation* – Necrosome members RIP1/pRIP3/pMLKL.

◼ *Native PAGE* – oligomerization of MLKL using anti-MLKL and/or anti-pMLKL antibodies.

Flow cytometric:

◼ *Non-fixed cells* – Cell permeable effector caspase (Caspase 3/7) activity probes (-), cell-impermeant DNA binding dyes such as propidium iodide (PI) (+), pMLKL (+). Cells that fall into the Caspase3/7 probe (-), PI (+), and pMLKL(+) gate are necroptotic.

◼ *Fixed cells* – Surface staining for pMLKL(+) before fixation followed by intracellular staining for TUNEL and cleaved Caspase 3/7.

### 2.4 Execution of necroptosis

Once assembled, the RIPK1-RIPK3 core necrosome recruits the ultimate executioner of necroptosis, namely the mixed lineage kinase domain like pseudokinase (MLKL). Canonical activation of MLKL occurs when activated RIPK3 with its kinase domain interacts with the pseudokinase domain of MLKL and phosphorylates it at Thr357 and Ser358 (20). After the critical phosphates are added to MLKL, it forms homotetramers, and prior to its release from the necrosome, two MLKL tetramers assemble to form homo-octamers, a process that does not depend on intramolecular disulfide bonds (21, 22). The oligomerization process of MLKL is a hotly debated topic, and although earlier work by Liu et al. using mouse and human cell lines provided evidence that MLKL tetramerization is a conserved event, newer evidence suggests otherwise (67). For instance, Petrie et al. discovered that RIPK3 phosphorylation of MLKL causes toggling of the pseudokinase domain activation loop, which promotes the necroptosis-inducing tetramerization of only human, but not mouse MLKL, suggesting species-specific differences in MLKL tetramerization (68). The molecular chronology of these events is still being investigated as exemplified by a recent study implicating a role of the TAM kinase family in promoting MLKL-mediated necroptosis via additional phosphorylation of MLKL at Tyr376 (69). The authors showed that inhibition or the absence of the TAM kinases prevents the progression of MLKL-mediated necroptosis and that TAM kinase activity is essential for the oligomerization step following necrosome release (69). However, further investigation is needed to determine the exact mechanism, the cells affected, and the critical dependence of necroptosis execution on the TAM kinase family. Moreover, octameric MLKL then forms Congo red dye-stainable amyloid fibrils of 5nm diameter, which are associated with necroptosis execution. However, it is not clear whether necroptosis execution critically depends on these MLKL amyloid-like polymers (23). One of the proposed mechanisms for oligomeric MLKL to execute cell death is its insertion into the membrane via its N-terminal helix bundle domain, which binds to the polar head groups of phosphatidylinositol phosphate in the membrane, creating a cationic large membrane pore (24, 25, 31). However, the exact mechanism of MLKL membrane insertion is currently under intense investigation and it is so far unclear how this process is exactly carried out. The membrane pore created by MLKL can cause the leakage of intracellular molecules of 10-kDa, leading to disruption of membrane integrity, osmotic imbalance, cell swelling, and ultimately lytic cell death (20). Thus, necroptotic cells are characterized by distinct changes in the cell morphology, cytosolic and nuclear swelling, and an inability to expel or prevent the influx of membrane-impermeable DNA binding dyes such as propidium iodide. However, these features alone are not sufficient to definitely diagnose necroptosis, and we therefore refer the reader to **Box 2** for an overview of the methods and tools that enable the proper detection of necroptosis. The readers should note that the methods for necroptosis detection, especially *in vivo*, have several limitations. The closest proxy for determining necroptotic cell death is the simultaneous labelling of phosphorylated MLKL and cleaved executioner caspases. It is still unclear whether necroptotic cells can be reliably labelled for double-stranded DNA breaks (DSBs) using the terminal deoxynucleotidyl transferase dUTP nick-end labeling reaction. Some data indicate that late necroptotic cells acquire DSBs by an unknown mechanism. However, the lack of caspase activation indicates that cells at early stages of necroptosis would not have detectable DSBs. Another common way that necroptosis is inferred is by showing that the cell death in question is blocked by necrosulfonamide. This method suffers from a major drawback, as necrosulfonamide also inhibits gasdermin D oligomerization and insertion in the plasma membrane and as such a non-canonical gasdermin D cleavage remains a possibility. Besides these, in long-term live cell imaging experiments, incubation of cells with caspase activity probes is associated with reduced caspase availability on cognate targets and may lead to experimental artefacts. Similarly, long-term incubation with PI is also associated with undesirable adverse effects on cell viability and must be considered.

### 2.5 Transcriptomics reveal cell type susceptibility to necroptosis

**Figure 1:**
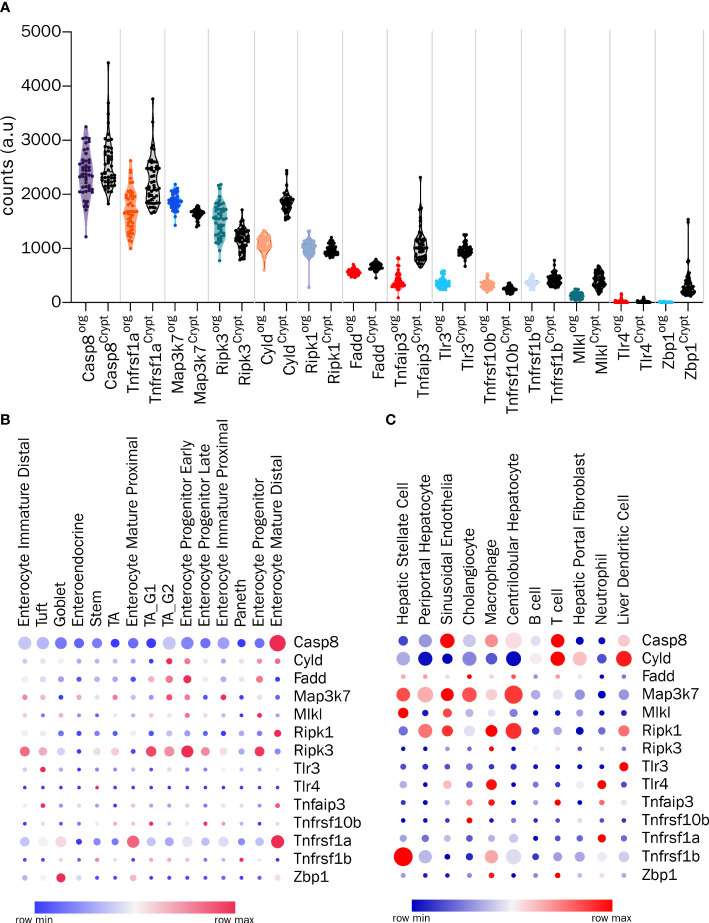
Transcriptomic analysis of the components involved in cell death in GI cells **(A)** Analysis of bulk transcriptomes from crypts and organoids for the indicated genes from GSE169368. **(B)** Expression profiles of indicated genes from single-cell RNA-Seq of intestinal epithelial cells (GSE92332) and **(C)** Expression profiles of indicated genes from single nucleus RNA-Seq of hepatic cells (GSE184506). In **(B, C)**, circle color indicates the scaled mean expression, and the size indicates percent of cells expressing the transcript.

Several components of the necroptosis machinery are expressed in the cells of the GI tract. With the sweeping advances in the ease of performing of bulk and single cell transcriptomic analyses, it is now possible to assess the relative expression of the various molecular mediators of cell death in a cell type and organ specific manner. In **Figure 1A** we have summarized the expression levels of the key regulators of necroptosis in the outstanding study by Lu et al. comparing the bulk transcriptomes of freshly isolated mouse intestinal crypts and intestinal epithelial organoids (70). It is clear that some of the components such as *Zbp1*, which is expressed in the crypts, are barely detectable in the organoid cultures, suggesting a non-epithelial origin of expression in fresh crypts. The levels of *Cyld* and *Tnfaip3* (gene encoding A20) were also lower in the organoids compared to the crypts (**Figure 1A**) (70). The only gene that tends to show slightly higher levels in the organoids is *Ripk3*, whereas the levels of *Tlr4* are barely detectable in either organoids or crypts (**Figure 1A**) (70). Interestingly, the single cell transcriptomic survey of the mouse small intestine by Haber et al. also confirms the overall relative abundance of the molecules associated with the cell death machinery, with the highest expression detected for *Casp8* and *Tnfrsf1a* (**Figure 1B**) (71). More importantly, the study allows the relative abundance of these transcripts to be examined in a cell type-specific manner. For example, as shown in **Figure 1B**, the expression of *Casp8* and *Tnfrsf1a*, is the highest in mature enterocytes, whereas that of *Ripk3* is the highest in early enterocyte progenitors (71). Only a small percentage of gut epithelial cell subsets express *Zbp1*, and among these, goblet cells are the highest expressers (**Figure 1B**) (71). On the other hand, the study by Nault et al. has revealed the single cell transcriptomes of mouse liver, allowing the exploration of the relative abundance of cell death associated molecules in different liver cell subsets (72, 73). As shown in **Figure 1C**, both centrolobular and periportal hepatocytes barely express any *Ripk3* (72, 73). This is consistent with reports showing that methylation and silencing of the *Ripk3* locus in hepatocytes renders them resistant to necroptosis even under conditions of immune and pathogen related stress (28, 29). Interestingly, hepatocytes express *Map3k7* (TAK1) and *Ripk1* (**Figure 1C**) (72, 73). These studies have shown how single cell transcriptomics can help to identify the susceptibility of specific GI cell types to necroptosis. Therefore, a pre-screening based on single cell transcriptomics followed by a systematic analysis using multiple molecular and cellular approaches will allow the identification of subtype-specific susceptibility to necroptosis in the future. Nevertheless, the readers are advised to interpret the results of the transcriptomic studies with caution given that transcript to protein expression correlations are especially poor in intestinal tissues (74).

## 3 Necroptosis in preclinical models of GI diseases

### 3.1 Intestinal models with an involvement of necroptosis

Several studies have highlighted the delicate balance of different cell death cascades in IECs by conditional ablation of these molecules in mouse IECs, which, when disrupted leads to overt intestinal inflammation and potentially life-threatening systemic sepsis (26, 27, 75–78). These studies collectively highlighted the essential pro-survival functions of NF-κB in the IECs, provided the first insights into RIPK1 and FADD as critical regulators of IEC apoptosis and RIPK3-driven necroptosis, and demonstrated the importance of the initiator caspase, caspase-8 in regulating apoptotic cascades in IECs, the failure of which triggered RIPK3-dependent IEC necroptosis. These studies also laid the foundation for the detection, analysis and study of apoptotic and necroptotic cell death in IECs. Inspired by these studies, several more recent studies have established the roles of other key regulators of IEC necroptosis, further utilizing *villin* promoter-driven *Cre* recombinase expression in mouse IECs as preclinical models. In their 2018 study, Kattah et al. demonstrated the critical role of A20 and ABIN3 in IECs downstream of the TNFR in suppressing the activation of caspase-8 and RIPK1. Deletion of ABIN3 and A20 in IECs led to spontaneous cell death of IEC organoids, demonstrating that this is an effect intrinsic to IECs. The authors were able to completely rescue the cell death phenotype by inhibiting RIPK1 or deleting RIPK3 in conjunction with caspase inhibition (79). These findings were later extended further to show the ability of ABIN3 to coordinate with A20 to regulate the K63 deubiquitination and subsequent activation of RIPK3 (80).

In an interesting study, using the inducible version of the Cre recombinase (CreERT2) downstream of the *villin* promoter, Wang et al. showed how an IEC-specific deletion of the histone methyltransferase *Setdb1* leads to a reactivation of endogenous genomic transposable elements (81). Reactivation of these dormant retrovirus-like elements triggered genomic instability in IEC stem cells and a ZBP1-mediated licensing of RIPK3 to induce necroptosis and intestinal inflammation. Pharmacological inhibition of RIPK3 was sufficient to cure the inflammatory phenotype of mice with the *Setdb1-*deficient IECs (81). Similar to viral dsDNA, intestinal ischemia-reperfusion injury is also associated with massive cell death via necroptosis and the release of mitochondrial DNA (mtDNA) into the tissue (82). The potential of mtDNA to induce necroptosis was directly addressed in a study by Zhang et al. The authors showed that a single intraperitoneal injection of mtDNA was sufficient to induce phosphorylation of both RIPK3 and MLKL, which could be inhibited by the administration of necrostatin-1 or by knocking out the gene *Sting*, a canonical DNA-sensor (83). Since the progression of necroptosis is critically dependent on the insertion of MLKL into the plasma membrane via its binding to membrane phospholipids, it is conceivable that altering the membrane lipid composition would have an impact on necroptosis. This speculation was supported by a recent study that examined the effect of IEC-specific deletion of the rate-limiting enzyme, cytidine triphosphate: phosphocholine cytidylyltransferase-α (CTα), which controls the synthesis of phosphatidylcholine (84). Inducible and conditional deletion of the CTα encoding gene *Pcyt1a* rendered mice susceptible to spontaneous colitis, increased number of TUNEL-positive cells in the colon, and increased RIPK3 mRNA and protein levels (84). Electron microscopy revealed characteristic features of necroptosis in both colonocytes and goblet cells (84).

The cognate physiological role of MLKL has been the subject of intense investigation, and studies have shown that MLKL may be involved in the regulation of autophagy upon its translocation to intracellular membranes (85). There is also evidence that lipidation of MLKL by very long chain fatty acids is critical for its function, although the mechanism is unknown (86). In an attempt to investigate the functions of the autophagy-associated protein ATG16L1 involved in LC3 lipidation, Matsuzawa-Ishimoto et al. uncovered an unexpected link of this autophagy regulator with IEC necroptosis. Using organoids generated from *Atg16l1* sufficient and deficient IECs, the authors showed that ATG16L1 inhibits TNF-driven necroptosis by regulating mitochondrial homeostasis (87).

Interestingly, a non-mouse model has recently been reported by Liu et al. showing that necroptosis is active in an LPS-TLR4-induced inflammation model in piglets (88). The authors used the detection of phosphorylated MLKL and the mitochondrial and nuclear alarmins PGAM5 and HMGB1 as readouts after a sub-lethal dose of LPS; however, it should be noted that this is not an IEC-specific model. Another study by Wen et al. used a non-mouse model of intestinal ischemia-reperfusion injury to demonstrate the involvement of necroptosis, as both histological injury and alarmin release were attenuated by the administration of necrostatin-1. In addition, the authors were able to show lower levels of RIPK1-associated MLKL by immunoprecipitation, suggesting the involvement of necroptosis in this model. However, just as with the piglet model, it should be noted that this is not an IEC specific model (82). In addition, studies relying solely on necrostatin-1 should be interpreted with caution since it also blocks and inhibits indoleamine-2,3-dioxygenase, an enzyme that modulates inflammation (89).

The most commonly used mouse model to understand human intestinal inflammation is the dextran sulfate sodium-induced colitis model. Interestingly, a study by Zhang et al. using mice that lack MLKL showed that the IEC loss seen in the DSS model appears to have some contribution from necroptosis, as MLKL ablation was protective against DSS in wild type mice (90). Another recent study supports the involvement of necroptosis in DSS-induced colitis, since the authors showed that GAB1 is involved in RIPK3 dephosphorylation and inhibition of IEC necroptosis using a DSS colitis model (91). However, Alvarez-Diaz et al. challenged these results in their report showing that germline deficiency of MLKL or RIPK3 exacerbates the severity of DSS-induced colitis (92). Therefore, the DSS colitis model is not an exclusive necroptosis inducing model per se. However, in combination with the genetic ablation of key necroptosis blockers such as *Casp8*, this model shows a high involvement of necroptosis in the IECs (59). Some studies have investigated the potential involvement of necroptosis using the pharmacological inhibitor of MLKL, necrosulfonamide (NSA), in the 2,4,6-trinitrobenzenesulfonic acid (TNBS)-induced mouse model of colitis (93). However, these studies must be interpreted in light of the promiscuity of NSA, which also blocks gasdermin D-mediated pyroptosis direct inhibition of gasdermin D or via a proximal modulation of caspase 1 activity (94, 95).

Future studies systematically analyzing the contribution of necroptosis in commonly used models of intestinal inflammation, including DSS colitis, TNBS colitis, oxazolone-induced colitis, and adoptive T-cell transfer-induced colitis, are warranted.

### 3.2 Hepatic models with involvement of necroptosis

An initial report on the potential involvement of necroptosis in the liver came from a mouse model of vaccinia virus infection (96). The authors showed that in several organs including the liver, vaccinia virus infection induces a phosphorylation-dependent RIPK1-RIPK3 complex and that the formation of the complex is necessary for necroptosis of the infected cells and thus infection control (96). They found that RIPK3-deficient animals had impaired infection control and increased inflammation. However, the study did not identify the cell types in the liver that underwent necroptosis. Later, Upton et al. revealed that viral factors can also inhibit RIPK3-dependent necroptosis in multiple tissues, including the liver, in a murine cytomegalovirus infection model (97). However, in contrast to the strong evidence for necroptosis in IECs in intestinal models, the role of necroptosis in hepatocytes has been a matter of debate. The first studies suggesting the involvement of necroptosis in hepatocytes used the acetaminophen-induced liver injury model (98, 99). Using RIPK3 silencing or genetic ablation in the acetaminophen-induced liver injury model and primary hepatocytes, the authors concluded that suppression of RIPK3 ameliorated liver injury, implicating hepatocyte necroptosis (98). After this report, other studies also showed an improvement in acetaminophen-induced liver injury upon RIPK1 inhibition with necrostatin-1 (99). Using another infectious hepatitis model of the hepatocyte-infecting bacterium *Listeria monocytogenes*, Koschel et al. uncovered an important role for the deubiquitinating enzyme, OTU domain aldehyde binding-1 (OTUB1) in the regulation of liver necroptosis (100). Under normal circumstances, an infection of this nature results in profound hepatocyte apoptosis, however, mice with a conditional ablation of OTUB1 in liver parenchymal cells showed a shift from apoptotic to necroptotic cell death. The authors inferred necroptosis from reduced levels of activated caspases 3, 7, and 8. Furthermore, the lethal infection phenotype that was observed in mice lacking OTUB1 in the parenchymal cells was rescued by the administration of necrostatin-1s and by co-ablation of MLKL (100). Thus far multiple studies had indicated the existence of necroptosis in the liver, albeit most suffered from an interpretation bias in which off-target pharmacological effects such as necrostatin mediated inhibition of RIPK1-driven apoptosis or necrosulfonamide-mediated inhibition of pyroptosis were not considered. Another confounding factor was with the interpretation of hepatocytes undergoing necroptosis, although germline knockouts of MLKL and not hepatocyte specific knockouts were used. Therefore, the question of whether hepatocytes are the targets of necroptotic cell death in hepatic pathology remained unanswered. Adding to this conundrum, a recent study by Preston et al. showed that both mouse and human RIPK3 promoter are epigenetically silenced in hepatocytes, thereby sparing hepatocytes from MLKL-mediated necroptosis (29). The authors used multiple models of infectious hepatitis and showed that the silencing of the RIPK3 promoter in hepatocytes was not relieved under any conditions of immunological stress tested in their study, deepening the mystery of hepatocyte necroptosis further (29). This hypothesis has been supported by studies investigating the association between transplant-related ischaemia and reperfusion-induced liver injury and necroptosis. In rats, alanine and aspartate aminotransferase levels were elevated after liver transplantation, clearly indicating hepatocyte damage. Interestingly, however, an evaluation of potential necroptosis by immunohistochemistry revealed increased levels of phosphorylated MLKL protein only in periportal cholangiocytes, but not in hepatocytes, suggesting that hepatocytes were spared from necroptosis, but not from other forms of cell death (101). The findings of this study are consistent with another study where the authors reported a dominant role of effector caspases in suppressing RIPK1-dependent necroptosis in this model (102).Yet, further reports have emerged which implicate ischemia-reperfusion-related liver injury in the context of aging livers to be dependent on hepatocyte necroptosis and ER stress (103, 104). A separate study confirmed this showing elevated levels of phosphorylated RIPK1, RIPK3, and MLKL in aged livers compared to young livers. The levels of total MLKL protein and its tetramer and octomerization as determined by native PAGE were also significantly increased (104). However, the authors could not conclusively show that this was a hepatocyte-specific increase in necroptosis.

Other studies have also implicated hepatocyte necroptosis in different mouse models of liver disease. In one such report the authors used ethanol-induced liver and hepatocyte injury. In this model, the authors reported a direct RIPK3-FADD interaction downstream of TNFR and an obligate involvement of TNF in driving the liver phenotype. Although, similar to the drawbacks of the previous studies, even this study used germline RIPK3 knockout mice, which afforded protection from the alcohol-induced liver pathology. However, the authors failed to rescue the liver phenotype by pharmacological RIPK1 inhibition, raising speculations of a RIPK1-independent activation of RIPK3 in hepatocytes (105). Another study implicated hepatocyte necroptosis in a rat model of ischemic acute kidney injury, which causes liver damage. The authors inferred hepatocyte necroptosis based on electron microscopic evaluation, and immunohistochemistry for RIPK3, identifying dying hepatocytes with features of necroptosis (106). However, a careful dissection of the specific cell types undergoing necroptosis using genetic models was not conducted.

In an interesting study using parenchymal-specific Cre driver line to ablate *Casp8*, Gautheron et al. performed a direct evaluation of necroptosis in liver parenchymal cells using a mouse model of methionine-choline dietary deficiency (MCD) of non-alcoholic steatohepatitis (NASH) (107). The authors showed that the *Casp8* deficiency in the parenchymal cells exacerbated MCD diet-induced liver injury and upregulated RIPK3 protein levels. Under these circumstances, when the authors co-ablated RIPK3, the liver phenotypes were rescued. Interestingly, the authors still observed a high level of CASPASE3 cleavage and activation in the liver demonstrating that MCD- triggered cell death in the liver could be dominated by RIPK1-depedent or classical apoptosis apoptosis in the absence of Caspase-8, via intrinsic pathways of effector caspase activation (107). In addition, the study failed to conclusively distinguish hepatocyte versus cholangiocyte necroptosis.

One of the direct evidences of necroptosis in hepatocytes was presented by Zhang et al. while uncovering an interesting role of O-linked β-N-acetylglucosamine (O-GlcNAc) modification in hepatocytes. Zhang et al. reported that the liver-specific conditional deletion of the O-GlcNAc transferase (OGT) gene causes hepatocyte necroptosis. The authors showed that primary hepatocytes with conditional ablation of OGT exhibited elevated levels of phosphorylated MLKL and cell death, which could be suppressed by treating the hepatocytes with GSK-872, a RIPK3 inhibitor, but not with z-VAD, a pan-caspase inhibitor suggesting a role for necroptosis in this mouse model (108). This study provided compelling *ex vivo* evidence that hepatocyte necroptosis can occur, however, potential off-target effects of GSK-872 were not considered and rescue using silencing of MLKL was not conducted.

In addition to these models, two other models of immune-mediated liver injury, namely concanavalin A (Con A) and α-galactosylceramide (α-GalCer)-induced injury have been widely used to investigate the mode of cell death in the liver (109). The ConA model is initiated by direct activation of CD4+ T- cells and NKT cells, which then target the hepatocytes in a manner that is independent of antigen presentation and TCR activation. On the other hand, the α-GalCer model is a milder, non-lethal model, that is predominantly an NKT cell-driven injury model in which T-cell involvement can be excluded (109). Using the Con A model and applying it to germline RIPK3-deficient mice, Deutsche et al. showed a mild degree of protection from liver injury, but not from lethal inflammation (110). Another study investigating the potential role of necroptosis in the Con A model also used RIPK3 knockout mice and found no significant differences in the extent of liver damage between the groups (111). Interestingly, when Gunther et al. applied the Con A model to mice deficient for the MLKL, they observed significant protection against liver injury (112). However, germline deficiency of RIPK3 gene could not protect against the Con A-induced liver injury. No detectable levels of RIPK3 were found in hepatocytes under any of the experimental conditions, raising the speculation of a RIPK3-independent mode of MLKL activation in hepatocytes (112). Similar to the caveats of the previous studies, no conclusive interpretation of hepatocyte necroptosis could be drawn based on these studies that have heavily relied on pharmacological inhibition and germline ablation.

Recently however, Hamon et al. reported that the hepatocyte cell death observed in the Con A model is non-necroptotic in nature, as mice with conditional ablation of MLKL in hepatic parenchymal cells were not protected from Con A-mediated liver injury and inflammation in their studies (28). In addition, some supporting evidence has emerged from the α-GalCer-induced liver injury model in the RIPK3 and MLKL deficient mice also with contrasting findings (113, 114). Therefore, the most plausible hypothesis at present is that necroptosis does occur in the liver during various pathophysiological conditions. However targeted approaches indicate that hepatocytes could be spared from canonical necroptosis owing to their failure to express RIPK3. Future investigations using targeted approaches will be necessary to prove the existence of potential novel mechanisms of necroptosis in hepatocytes. In summary, while new findings indicate that immune-mediated necroptosis mainly occurs in non-parenchymal cells of the liver, the precise role of necroptosis in diseases of the liver remains under debate.

### 3.3 Pancreatic models with involvement of necroptosis

Cell death modes in pancreatic pathology have been intensively investigated, particularly in the context of pancreatic islet β-cell death. Transplantation of donor islets into recipient animals is often associated with increased host-versus-graft immunity and associated death of cells in the transplanted tissue. Zhao et al. used a model of CD4^+^ T-cell mediated autoimmune diabetes after transplantation of donor islets with altered expression of the proteins involved in the cell death machinery to investigate the role of different modes of cell death (115). Here, the authors assessed the autoimmune attack on islets upon adoptive transfer of FACS sorted CD4^+^ BDC2.5^high^CD25^-^ diabetogenic T-cells into recipient mice. The authors showed that islets from RIPK3-deficient recipient mice were still equally susceptible to the autoimmune T-cell- mediated destruction (115). Moreover, recipient mice with a concomitant inhibition of both RIPK3 and BLC2 to block necroptotic and apoptotic cell death were still unable to protect the islets from the autoimmune attack (115). These results seem to be in line with a report that described rare MLKL mutations in individuals, which were associated with an increased risk of maturity-onset diabetes of the young potentially acting as an epistatic modifier of existing disease mutations in the PDX1 gene (116). The authors showed that the mutant form of MLKL was unable to induce necroptosis; however, the patients still developed diabetes and beta cell loss potentially via other modes of cell death, arguing against a role for necroptosis in β-cell loss (116). The induction of pancreatic β-cell death and the consequent diabetes in mice and rats is achieved by five consecutive intraperitoneal doses of streptozotocin (STZ). Between two and five days after STZ administration, mice begin to show reduced circulating insulin levels and increased glucose levels due to β-cell death (117, 118). A few studies have investigated the involvement of necroptosis in this model. For example, Contreras et al. showed that isolated pancreatic islets from the RIPK3 knockout mice were protected from TNF- and z-VAD- induced cell death compared to control islets (33). The authors also observed a significant reduction in the STZ induced hyperglycemia and glucose intolerance in mice with a germline deficiency of RIPK3. The authors concluded that in this model, the TNF-induced RIPK1-RIPK3 pathway is operational and STZ- induces necroptosis of β-cells (33). Taken together, the majority of studies indicate that islet cells of the pancreas are resistant to necroptotic cell death under conditions of autoimmune attack, however, a detailed molecular and biochemical characterization of the mode of cell death is still missing. Therefore, RIPK3-dependent necroptosis as a contributing factor to autoimmune islet cell death can be excluded for the time being. Unlike the autoimmune destruction of islets in diabetes, pancreatitis is frequently associated with a loss of acinar cells, a feature recapitulated in the mouse model of cerulein-induced acute pancreatitis. Using RIPK3 knockout mice, Zhang et al. and He et al. independently showed a crucial role of necroptosis in driving acinar cell loss, and tissue damage in the cerulein-induced pancreatitis model (119, 120). This was followed by the report by Louhimo et al. who showed a direct induction of MLKL phosphorylation in pancreatic acinar cells at supramaximal concentrations of cerulein or submicellar concentrations of the bile acid taurolithocholic acid-3-sulfate (34). The authors also reported an improvement in pancreatitis upon RIPK3 ablation or pharmacological inhibition of RIPK1 via necrostatin-1 (34). Another study by Zhao et al. employed the cerulein-induced acute pancreatitis model, in RIPK3 knockout mice as well as in mice carrying a mutation in the kinase domain of RIPK3, rendering it catalytically inactive. The authors showed a significant reduction in the number of dead pancreatic cells, again implying that the kinase function of RIPK3 was critical in mediating acinar cell loss in this pancreatitis model (121).

In contrast to these studies that clearly linked RIPK3-mediated necroptosis with cerulein-induced pancreatitis, a report by Newton et al. found no contribution of necroptosis to pancreatitis severity in histological and serological endpoints (122). The authors showed that in mice bearing a mutation in the RIPK1 kinase domain a germline deficiency of the RIPK1 kinase domain or of RIPK3 were not rescued from cerulein-induced pancreatitis. In addition, a recent report by Boonchan et al. provided more evidence against the involvement of necroptosis in acinar cell loss in acute pancreatitis (123). The authors used knockouts for both RIPK3 and MLKL and showed that RIPK3 deficiency resulted in a more severe pancreatic inflammation and disease, and MLKL deficiency rendered mice even more susceptible to tissue damage caused in the cerulein-induced acute pancreatitis model. Their findings showed that the loss of the key executioner of necroptosis shifted the balance of cell death towards apoptosis via a downregulation of *Bclxl* and *Cflar*, detectable as an increase in the number of TUNEL positive apoptotic cells (123). However, the authors did not investigate whether pharmacological pan-caspase blockade could mitigate the increase in disease severity ascribed to increased apoptosis. Several other studies have used the cerulein-induced acute pancreatitis mouse model to investigate necroptotic cell death in the pancreas (124, 125). However, given the conflicting results of some of the systematic investigations, we recommend that this model be used with caution when interpreting or implicating necroptosis as a driving factor until conclusive evidence on the role of necroptosis in the pancreas becomes available.

In summary, there is biochemical and genetic evidence showing that pancreatic acinar cells express the key components of the necroptotic machinery, but there are significant discrepancies regarding the evidence for necroptotic cell death in pancreatic pathology. The discrepancies may emerge from the different treatment times and concentrations used in these studies to induce acute pancreatitis. Although blocking RIPK3 seems to lower pancreatitis severity, the contribution of necroptosis-independent functions of RIPK3 needs to be investigated. Further research using targeted conditional and inducible knockout mice for various components of the necroptosis machinery is needed to fully address the role of necroptosis in pancreatitis.

## 4 Therapeutic discovery: necroptosis interventions

### 4.1 Translation of RIK1 Inhibitors, from bench to bedside

As our understanding of the molecular cascade and signaling involved in necroptosis has improved, the prevention of necroptotic cell death has become a therapeutically viable opportunity. This is particularly true in case of diseases where overt and excessive cell death occurs triggering further inflammation and tissue damage. Therefore, small molecule inhibitors to block necroptosis and enhance cytoprotection are being investigated for potential applications in organ preservation for transplantation, autoimmune diseases, and viral infectious diseases. As therapeutic targeting of necroptosis has evolved, three clear targets have emerged: 1. RIPK1, 2. RIPK3 and 3. MLKL. A kinase inhibitor screen identified several type I inhibitors that competitively inhibit the ATP binding site of the RIPK1 (126). Interestingly, several of these inhibitors are FDA- approved kinase inhibitors, such as sunitinib, that block a wide variety of kinases other than RIPK1 and are therefore not selective/specific (**Table 2**). Other compounds, such as the FDA-approved drug pazopanib, as well as compounds from the GSK kinase inhibitor library, have been identified as type II inhibitors that bind to RIPK1 in an inactive kinase conformation (DFG-out), sterically blocking the ATP binding site (127, 128). Unfortunately, further development of these type II inhibitors was stalled due to higher molecular weights, lower solubility in aqueous solvents, and several off-target kinase inhibitory effects (128). Finally, the necrostatin class of inhibitors has also proven to be a useful platform for pharmacological development of RIPK1 inhibition. Necrostatins bind to RIPK1 in its inactive conformation via the nitrogen atom in the indole ring of necrostatin, forming an H-bond with a hydroxyl group in the activation loop of RIPK1 (127, 129, 130). Several derivatives of necrostatin-1 have been developed, and protect preclinical mouse models of colitis, pancreatitis and steatohepatitis. However, like necrostatin-1, they suffer from poor pharmacokinetics, lack of efficacy, and off target effects due to the inhibition of other enzymes, particularly indoleamine-2,3-dioxygenase, which have critical functions in immune cells (131). Recently, a randomized, placebo controlled clinical trial of a new first-in-class RIPK1 kinase inhibitor, GSK2982772, in active ulcerative colitis was completed. The compound has better pharmacokinetic properties, higher potency and selectivity, and was well tolerated with no safety concerns (128, 132). Interestingly, at the 43-day trial mark, the GSK2982772 group had significantly lower fecal calprotectin levels and a modest reduction in mean circulating CRP levels compared to the placebo group. However, these differences disappeared at later time points and no demonstrable improvements in efficacy were observed in terms of improved Ulcerative Colitis endoscopic index of severity (132). Considering the short period of time during which improved levels of fecal calprotectin and CRP were observed, together with the recent observation of a stage-dependent effect of RIPK1 inhibition on inflammation, it would be necessary to identify the optimal therapeutic window of inhibition. Another recent discovery has raised the possibility of a co-regulatory core molecular platform called the PANoptosome which controls multiple inflammatory modes of cell death that has been termed “PANoptosis”. The core feature of panoptosis is the activation of RIPK1 by extracellular pathogens or danger signals, or of ZBP-1 via intracellular pathogens or danger signals. The potential therapeutic value of targeting PANoptosis in inflammatory diseases has also been recently assessed against severe COVID-19 given that SARS-CoV-2 increases RIPK1 activation and ZBP-1 expression to promote viral spread (133–135). The authors showed that human lung organoids infected with the SARS-CoV-2 virus had a lower viral load and reduced inflammation upon treatment with Nec-1s (134).

**Table 2:** Therapeutic and experimental inhibitors against necroptosis.

Target	Inhibitors	
	Non-selective	Selective
RIPK1	Sorafenib, Sunitinib, Pazopanib, Tozasertib, Ponatinib, Necrostatin1, Necrostatin1s,	GSK2982772, GSK3145095, DNL747
RIPK3	Sorafenib, Ponatinib, Pazopanib, Dabrafenib, Necrostatin 1, GSK′840, GSK′843, GSK’067, GSK’074	GSK2399872A
MLKL	Necrosulfonamide	

### 4.2 Potential of RIPK3 inhibitors

Few type I inhibitors of RIPK3 have entered therapeutic development, mainly due to the identification of toxic kinase-dead variants in which the aspartate in the conserved DFG motif is replaced by asparagine (136). This substitution resulted in uncontrollable RIPK1- and caspase-8- dependent apoptosis and midgestational death, raising safety concerns of overt apoptosis induction upon the inhibition of RIPK3 kinase functions (131, 136). Interestingly, however, the FDA-approved ATP-competitive BRAF inhibitor dabrafenib also inhibits human and mouse RIPK3 without cross-inhibition of RIPK1 and without adverse induction of RIPK1-dependent apoptosis and protects mice from acetaminophen-induced liver injury (137, 138). However, other type I inhibitors of RIPK3, such as GSK’872 and GSK’843, do not spare RIPK1-FADD-CASP8 complex formation and apoptosis induction, and therefore therapeutic exploration has been inconsequential (131). Interestingly, a recent study reported the effects of GSK’872 treatment in mice expressing human ACE2 in the nasal and lung epithelium infected with the SARS-CoV-2 viruses to inhibit the ZBP-1 branch of PANoptosis in a mouse model of COVID-19 (135). Treatment with GSK’872 significantly reduced MLKL phosphorylation, however, chemokine production remained unaffected. On the contrary, mice with a genetic ablation of RIPK3 displayed a significant reduction in chemokine levels upon SARS-CoV-2 infection compared against infected wildtype controls, implying that inhibition of the kinase functions of RIPK3 alone may not be sufficient to control inflammation (135). Several type II kinase inhibitors such as the FDA approved drugs ponatinib and sorafenib, which target RIPK1, also block the kinase activity of RIPK3 and can protect mice from TNF-induced systemic inflammatory response syndrome (SIRS) and renal ischemia-reperfusion injury (139). Attempts to develop newer RIPK3 inhibitors have met with some success. For example, Xia et al. recently identified Zharp-99, an unclassified small molecule kinase inhibitor of RIPK3, which blocked necroptosis in human and mouse cells without inducing RIPK1-dependent apoptosis and also blocked TNF-induced SIRS in mice (140). Interestingly, a recent study showed that blocking the oligomerization of RIPK3 can also be used as a new strategy to inhibit necroptosis without increasing apoptosis via the pharmacological antagonist of SRC, named 1-(tert-butyl)-3-(4-chlorophenyl)-1H-pyrazolo[3,4-d] pyrimidin-4-amine (also known as PP2). The authors found that PP2 specifically disrupted RIPK3 oligomerization in cell lines, without altering its autophosphorylation or its association with RIPK1 and MLKL (141). However, the *in vivo* safety, efficacy, and specificity of these newly developed compounds that block RIPK3 function remain a topic for further investigation.

### 4.3 MLKL as a potential future therapeutic target

Pharmacological inhibition of the final executioner of necroptosis, MLKL, is also promising but is still in its infancy due to the complications of targeting a pseudokinase where screening of well-established kinase libraries is not feasible. Mice carrying a germline mutation of MLKL are viable and show no apparent adverse phenotype at steady state (142). In addition, mutations in the brace region of MLKL, which generate a lethal constitutively active MLKL variant, are only toxic if homozygous, demonstrating that normal MLKL copies can induce clearance of toxic versions of MLKL (143). By screening a compound library of over 200,000 compounds in an anti- necroptosis cell screen, Sun et al. identified a small molecule called (E)-N-(4-(N-(3-methoxypyrazin-2-yl) sulfamoyl)phenyl)-3-(5-nitrothiophene-2-yl)acrylamide, which they named necrosulfonamide as a molecule that targets the Cys86 residue of MLKL (60). The authors showed that the targeting mechanism is specific to human MLKL, as the Cys86 residue is not conserved in mouse cells and therefore necrosulfonamide is unable to inhibit necroptosis in mouse cells (60). Recent evidence has challenged the specificity of necrosulfonamide by showing that in addition to MLKL-mediated necroptosis, necrosulfonamide also inhibits GSDMD-induced pyroptosis (144). Following this report, several studies showed that necrosulfonamide prevented disease outcomes in mouse models of diseases such as acute brain injury, colitis, acute liver failure, and myocardial dysfunction due to its pyroptosis inhibitory effects (144–147). Recently, a new class of xanthine and uracil derivatives has been reported to stabilize the inactive state of MLKL, thereby inhibiting MLKL oligomerization and membrane translocation, but their precise mechanism of action, *in vivo* safety, and off-target effects remain to be elucidated (148, 149). To the best of our knowledge, no clinical phase trials have been conducted for MLKL inhibitors so far. Future strategies of computational repurposing of approved drugs with the potential to cross-inhibit MLKL-mediated necroptosis, may achieve faster translational results (150).

## 5 Concluding remarks

A large body of evidence points to the role of necroptosis of various GI cells in the context of infectious and inflammatory diseases. The modeling of necroptosis in preclinical disease is driven by our growing understanding of the underlying molecular machinery and is fueled by conditional genetic and pharmacological screens. However, to date, the collective beneficial evidence from preclinical studies outweighs the translational efforts in the development of safe, efficacious, potent, and selective inhibitors, which target this cell death mechanism in human diseases. Future development of newer interventions, therapeutic strategies, and repurposing of existing drugs against necroptosis is needed.

## Author contributions

JP and CB: conceptualization and composition. MG: analysis. MB: editing and proofreading. All authors contributed to the article and approved the submitted version.
